# Cognitive interventions in children and adolescents from low socioeconomic status backgrounds: a systematic review protocol of randomized controlled trials

**DOI:** 10.1186/s13643-021-01738-x

**Published:** 2021-06-25

**Authors:** Rosalba Company-Córdoba, Antonio Sianes, Ian Craig Simpson, Joaquín A. Ibáñez-Alfonso

**Affiliations:** 1 Department of Psychology, Human Neuroscience Lab, Universidad Loyola Andalucía, Avda. de las Universidades, Dos Hermanas, 41704 Seville, Spain; 2 ETEA foundation, Development Institute of Universidad Loyola Andalucía, Córdoba, Spain; 3 Research Institute on Policies for Social Transformation, Universidad Loyola Andalucía, Córdoba, Spain

**Keywords:** Cognitive intervention, Cognitive development, Socioeconomic Status (SES), Poverty, Children, Adolescents, Systematic review, Protocol

## Abstract

**Background:**

Many studies have evaluated the effects that a lack of resources has in children’s physical and cognitive development. Although there are reviews that have focused on applied interventions from different perspectives, there is still a need for a comprehensive review of what has been attempted with these children from the cognitive intervention field. The aim of this paper is to present a protocol for a systematic review collecting randomized controlled trials (RCTs) studies whose purpose was to enhance cognitive development through the implementation of cognitive interventions in children and adolescents (< 18 years old) from low socioeconomic Status (SES) settings.

**Methods:**

The following databases will be searched: Web of Science (WoS core collection), PsycINFO, Cochrane Central Register of Controlled Trial, ERIC, PubMed, ICTRP and Opengrey Register (System for Information of Grey Literature in Europe). Searches will be adapted for each database. Additionally, the reference list of articles included in the review will also be searched. As part of this process, two reviewers will determine, independently, the suitability of each article taking into account predefined inclusion/exclusion criteria. Pertinent data will then be extracted, including sample characteristics, specifics of the intervention, and outcomes, as well as follow-up measures. Internal validity will be assessed using the Cochrane Risk of Bias Tool. A quantitative synthesis of results will be conducted using a meta-analysis. However, if a meta-analysis is difficult to implement due to the diversity of the studies (for example, in terms of measures used to estimate the effect size, intervention types, outcomes, etc.), the technique synthesis without meta-analysis (SWiM) will be used. A description of outcome measures will be provided, as measured by validated neuropsychological instruments of any cognitive function.

**Discussion:**

The systematic review will offer a framework based on evidence to organisations, institutions, and experts who want to implement or promote interventions aimed at enhancing cognitive domains in children and adolescents who live in disadvantaged contexts.

**Systematic review registration:**

This protocol was registered with the International Prospective Register of Systematic Reviews (PROSPERO) on 16 March 2020 (registration number: CDR42020150238).

**Supplementary Information:**

The online version contains supplementary material available at 10.1186/s13643-021-01738-x.

## Background

According to the United Nations (UN) [1], multidimensional poverty affects approximately 1300 million people around the world. Consequently, this number of people living with sub-optimal access to health services, educational opportunities, and daily life facilities needed to achieve a minimum level of well-being. Living in exclusion conditions has consequences related to quality of life in terms of lack of food, inadequate living conditions, and many other negative factors. Such conditions tend to support the perpetuation of poverty through successive generations [2]. Of those that suffer from multidimensional poverty, it is estimated that half are children [3]. Of note, childhood poverty is not a specific condition of developing countries considering that one out of five children living in developed nations experiences vulnerable conditions [4]. Childhood poverty is a complex phenomenon in that it is not just related to economic factors. It also involves elements that affect children’s well-being, such as lack of stimulation, overcrowded homes, lower parental education, and high levels of parental stress or anxiety [5], among others factors. Nevertheless, low familial Socioeconomic Status (SES) is associated with the emergence of childhood poverty. SES indicators refer to different factors such as family income, parent occupation, or parental education [6]. Family SES affects children’s lives in complex ways with the period of time living in poverty conditions being one important factor that impacts on their well-being [7]. In relation to this, many studies have shown how SES influences the adequate fulfilment of processes related to physical, emotional, and cognitive development [8, 9]. Additionally, low-SES is associated with malnutrition [10, 11]. According to the United Nations Children’s Fund (UNICEF) [12], globally, poor nutrition negatively impacts the development in one out of every three children. Worldwide, around 50 million children are considered underweight for their height and around 149 million children suffer from developmental delays [13].

Focusing on cognitive development, defined here as the maturational process through which children develop abilities and strategies to adapt themselves to changeable settings, it has been shown that living in poverty conditions can have a negative impact in cognitive domains [14]. Cognitive domains involve processes allowing an individual to perceive and organize internal and external information. These mental activities include perception, attention, memory, language, and executive functions. Executive functions have been widely studied [15] and encompass those mental activities related with information management and the exhibition of goal-directed behaviours: response inhibition, cognitive flexibility, working memory, planning, reasoning, self-monitoring, and the like. From previous literature, it is known that the brain systems which show the greatest anatomical and functional differences in children as a function of their SES are those related to language and executive functions [16–18]. The efficiency in managing these complex cognitive activities can predict aspects related with academic performance [19], which is a key factor in determining a child’s life outcomes. In relation to this, children who live in disadvantaged conditions tend to underperform in school in comparison with their wealthier counterparts [7, 20]. Consequently, these children suffer a disadvantage in their academic lives from an early age.

From a global perspective, international initiatives such as those proposed by the United Nations in the 2030 Agenda through the Sustainable Developmental Goals (SDGs) are aimed at eradicating the differences that poverty causes around the world [21]. In relation to this, and in order to help overcome the difficulties that children from low-SES have in their cognitive performance, a number of different interventions and programs have been proposed and applied. Due to the nature of the disadvantaged context, intervention processes can be influenced by many factors. The heterogeneity of these factors could make it difficult to establish a “gold standard” intervention that leads to meaningful improvements in the cognitive domains of these children. Nevertheless, it has been demonstrated that implementing multi-modular programs that include significant agents and contexts could benefit different cognitive processes as well as providing benefits in other important areas of these children’s lives, such as in emotional and academic domains [22–24].

Diverse intervention studies have been conducted in order to determine if the negative effects that low-SES settings have on children’s development can be alleviated. These interventions usually involved the children being assessed prior to the intervention and assessed for a second time once the intervention had concluded. This design enables improvements due to the intervention the children’s performance or behaviour to be identified. Accordingly, it is recommended that pre-post evaluations be carried out using standardized evaluation tools to allow the child’s performance to be compared with his reference group in terms of age, gender, etc. In this regard, cognitive interventions correspond to systematic actions designed and implemented by professionals in order to compensate or enhance cognitive functions. Furthermore, Bronfenbrenner [25] states that structured interventions designed to improve children’s cognitive skills may be considerably more effective compared with other less-structured interventions, especially when applied in a relevant context such as in the school curricula.

Of note, it is important to remember that at-risk children are influenced by many agents and context. Thus, a broad intervention program should include actions which target these diverse factors. These types of multi-modular programs include, for example, actions within the family context and educational initiatives, along with health-related interventions. A large number of such multicomponent intervention programs to improve disadvantaged children’s health and performance in, for example, cognitive and behavioural domains have been reported [22, 24, 26, 27]. Additionally, some researchers have studied the importance of implementing programs aimed at improving children’s development through indirect factors such as nutritional status [28–30], physical activity [31], and applying programs which target a specific cognitive domain [32].

Another important factor to consider is whether the implementation is individual, or is carried out in a group setting. The individual format is appropriate in cases in which the researcher or the clinician desires to specifically adapt the intervention to the person. Consequently, it is useful to look for subtle enhancements or nuances in the child’s responses. This kind of intervention requires a greater investment of time and material, along with human capital, in order to be totally centred on the child’s potential and necessities. In contrast, a group setting allows the intervention to be implemented in less time, and with a lower investment of personnel and materials. Moreover, group settings may resemble the school environment in which children are immersed in a daily-basis, and thus recreate a more familiar environment for the child. Within the literature, there is evidence of both individual [33] and group [34, 35] interventions applied in order to enhance vulnerable children’s cognitive processes. For example, Segretin et al. [36] compared both individual and group cognitive interventions applied to 4-year-old vulnerable children reporting that the children participating in the group setting improved their performance in memory and planning processes, whereas the intervention using individual sessions was more effective in terms of attentional outcomes.

Previous literature exists which aims to offer a comprehensive analysis of cognitive interventions applied to vulnerable children and adolescents. For instance, Hermida et al. offered a compendium of intervention programs based on developmental psychology and cognitive neuroscience [23]. These authors pointed out further considerations that may be important when designing an intervention program to promote disadvantaged children’s cognitive development, such as the importance of considering the developmental cognitive neuroscience and the need to work on basic cognitive processes. Moreover, in her synthesis, Warr-Leeper [37] offered a description of early intervention programs that have shown effectiveness in this at-risk population. This synthesis included a comprehensive review of well-known programs, the vast majority of which were conducted in the USA that aimed to alleviate the negative impact that living in disadvantages settings has for children’s development. Some examples of the programs reviewed are as follows: Head Start Program [38], Carolina Abecedarian Project [39], Project CARE [40], and Syracuse University Family Development Research Program [41]. These programs are characterised by the fact that they include diverse services in order to meet the children’s needs as completely as possible. Health services, parenting programs, and family support are some of the benefits that participating families were able to receive. Research studies conducted within the framework of these programs demonstrated improvements in the cognitive development of the children who participated, except in the case of the Syracuse University Family Development Research Program [41], in which children showed a better socioemotional performance, but did not show improvements in cognitive performance [37].

Furthermore, there are also systematic reviews of intervention programs whose goals were to enhance the development of children specifically from low-SES settings. These reviews have focused on different aspects, including parent-centred interventions [42], interventions designed around home-visits [43], home-based programs [44], programs focusing on improving nutrition [28], and interventions centred on a specific cognitive domain; for instance, language stimulation [45]. Moreover, a systematic review undertaken by Kathib et al. [46] suggested the importance of interventions implemented in early childhood in order to ensure a healthy development in low and middle-income countries. A very recent study conducted by Saitadze and Lalayants [47] has offered a compendium of mediation mechanisms that may modulate the negative effects that poverty-associated factors have in children’s cognitive and emotional outcomes. These authors have pointed out that having proper stimulating material and an adequate parental support could cushion the impact poverty has on childhood development.

Focusing on linguistic outcomes, Heidlage et al. [48] conducted a meta-analysis of parent-centred interventions in low-SES children. In contrast, the goal of Scionti et al.’s systematic review [49] was to assess the efficacy of cognitive training focused on executive functions in pre-schoolers with low-SES and ADHD. Furthermore, due to the important role that parents play in early childhood, some studies have promoted children’s development through parental intervention [45, 50].

Considering all of the factors in these studies shown to be associated with improving low-SES children’s development, it is not difficult to imagine the great heterogeneity of results within the field. The paediatric population includes a wide range of ages in which important changes are presumed to occur in different areas of children and adolescents’ lives. This is just one possible source of heterogeneity that may exist in the studies that aim to enhance cognitive development through interventions. Depending on the age and cognitive outcomes in which researchers want to specifically work on, interventional studies will potentially be completely different. Another possible source of variability in terms of methodologies is the design and implementation of the study, as interventions conducted in relatively wealthy regions or countries are likely to have more and better resources than studies conducted in developing regions. That is, the political and social contexts in which a study is implemented will almost certainly impact on many factors of the study [36].

With regard to methodologies, implementing a full and well-conducted randomized controlled trial (RCT) may be difficult to implement with disadvantaged populations. Although this type of design is recommended in order to determine the efficacy of an intervention, its implementation does involve some design considerations. RCTs require more time to implement, as well as more economic and human resources to be conducted (e.g., more research staff are needed to evaluate and implement the intervention to avoid bias). Furthermore, this type of study has ethical implications in the health area (e.g., to withhold effective interventions to participants in the control group in order to meet the research goals). Given the difficulties with low-SES environments previously described, some authors have used quasi-experimental designs [34, 35, 51, 52] to implement cognitive interventions, as implementing a full experimental design can be difficult.

Although the previously mentioned reviews provide valuable insight to advance the field, there are some limitations. For example, some reviews did not adhere to standardized guidelines [23] and others did not offer a comprehensive perspective focusing on interventions designed to enhance cognitive development in low-SES children [48]. Additionally, most of these reviews focus on the study of different types of interventions to enhance children cognitive development, but they did not focus on the interventions themselves. Consequently, since the authors of this protocol were not able to locate a systematic review that answers the research question: *which cognitive interventions have been used to enhance the cognitive development of children and adolescents from low-SES setting using a RCTs design?*, they decided to conduct a systematic review to fill this gap in the field.

### Objectives

The aim of this paper is to present a protocol for undertaking a systematic review collecting randomized controlled trials (RCTs) studies designed to enhance cognitive development through the implementation of cognitive interventions in children and adolescents (< 18 years old) from low-SES settings. The systematic review will offer an analysis of interventions that have been used depending on the cognitive domain, age, and sociocultural characteristics of the sample, along with their effectiveness.

## Methods

The Cochrane handbook for Systematic Reviews of Interventions [53] was used to design the protocol and the Preferred Reporting Items for Systematic Reviews and Meta-Analyses Protocols (PRISMA-P) [54] was followed to describe the protocol. The systematic review protocol was registered in the International Prospective Register of Systematic Reviews PROSPERO (http://www.crd.york.ac.uk/PROSPERO) on 16 March 2020 with the registration number CRD42020150238.

### Eligibility criteria

In order to answer the research question, the inclusion and exclusion criteria were established following the PICOS protocol [53]. The goal of this protocol is to ensure that only relevant studies are included in the systematic review. To achieve this, PICOS provides a set of criteria in 5 different areas, and studies must adhere to all of these guidelines to be included (Table 1). The Additional file 1 provides a summary of how the criteria in these 5 areas will be applied for the proposed protocol.

**Table 1:** How the PICOS eligibility criteria will be applied to the proposed protocol

Criteria	Inclusion	Exclusion
**Language**	English, Spanish	Other languages
**Year of publication**	All	-
**Population (P)**	Children and adolescents from 0 to 17 years old (both included)	Adults
**Intervention (I)**	Neuropsychological/Cognitive interventions	Any other type of intervention: emotional, social relations, solely family interventions, etc.
**Comparison (C)**	Any kind of comparator	-
**Outcome (O)**	Cognitive domains (perception, attention, language, memory, praxias, or executive functions)	Other domains (e.g., emotional-state, social relations, family relations)
**Study design (S)**	Randomized controlled trials	Prospective cohort, retrospective cohort, cross-sectional, case-control, systematic reviews and meta-analysis, protocols, clinical case, editors’ letters, qualitative studies and observational studies

#### Types of studies

In this case, studies which have used a randomized controlled trial design RCTs (individual and cluster randomized controlled trials) will be included. This type of design reduces the probability of bias by using the randomized assignment of participations to groups, so it is the most suitable design to determine the effectiveness of an intervention [54]. No other kind of methodology will not be considered (see Additional file 1).

#### Type of participants

Studies which have undertaken interventions with either children or adolescents (from 0 to 17 years old) will be included, regardless of gender. Furthermore, to be included in the revision, the context of the participants must be described by the authors as disadvantaged, vulnerable, or low-SES (e.g., disadvantaged families, neighbourhood or school, lack of resources). In cases where there is no explicit information about the sample’s socioeconomic status, the authors will be contacted in order to ascertain this information. There will be no restrictions in terms of geographic location. Studies that only include a clinical population (e.g., neurodevelopmental disorders, traumatic brain injury) will not be included in the systematic review—that is, at least one of the intervention groups must comprise healthy children in order to assess the effectiveness of the intervention in this population. No exclusion criteria regarding intellectual development will be applied.

#### Type of intervention

The systematic review will include those studies in which a cognitive intervention had been implemented, whether this be exclusively, or among other interventions. Studies whose primary or secondary intervention goal was the enhancement of individual cognitive capacities [55] through the implementation of a set of directed and planned activities to achieve significant improvements in one or more of the cognitive domains will be considered. The intervention or training in any specific cognitive function process (e.g., selective attention, working memory, vocabulary) will be included.

Interventions designed to benefit cognitive performance directly or indirectly which have been implemented with children will be included. If, due to their age, children are not sufficiently independent, parental interventions designed to improve the cognitive capacities of their children will also be considered valid. Studies that apply concomitant interventions (e.g., nutritional, social skills) will also be taken into account. Studies that exclusively include psychosocial, emotional, familiar, nutritional, or any other kind of intervention will not be included in the systematic review.

#### Types of comparators

Comparator groups will include any other kind of intervention which is not specified as a structured cognitive intervention—that is, interventions which have not been shown to have cognitive impact, or which have not included an active or passive control group.

#### Outcome measures

Reviewers will only consider RCTs studies in which a primary or secondary outcome is referred to as a cognitive performance measure. Included studies’ outcomes must have been assessed using suitable and validated neuropsychological tools in order to obtain the intervention effectiveness through a pre-post assessment design. When authors do not provide information about the cognitive assessment tool validity, the reviewers will conduct a literature search to determine the tool’s psychometric properties.

Included studies must report scores obtained with cognitive assessment tools, assessed through pre-post appraisal. The valid outcomes that can be assessed and can be the focus of interventions in the studies are those known in the field as cognitive domains. This concept has been widely described in the literature, being commonly accepted to include perception, attention, language, memory, praxias, and executive functions. The term “executive functions” refers to goal-directed high-order cognitive processes [15] which involve inhibitory control, working memory, cognitive flexibility, and reasoning. Outcomes that specifically correspond to a cognitive domain processes (e.g., sustained attention, vocabulary, visual perception) will be considered as valid. Other outcome measures will not be included in the systematic review.

### Search methods for identification of studies

#### Electronic searches

The literature search will be conducted in the following databases: Web of Science WOS core collection (1900 to present), PubMed (1975 to present), ERIC (1979 to present), Cochrane Central Register of Controlled Trial (all available dates), PsycINFO (1973 to present), the International Clinical Trials Registry platform, ICTRP (all available dates), and Opengrey (System of information on Grey literature in Europe). In this last database, only dissertations from 2018 to date will be included. The rationale for this is to identify information in recent dissertations which is as yet unpublished in scientific journals. The databases were chosen because of their compatibility with the topic of systematic reviews. Additionally, we will conduct a manual search in the references list of each selected article.

The search strategy will include terms extracted from the relevant literature that approximate our research question. An example of the used search strategy in one of the databases is included in Additional file 2. Reviewers will use terms related with the participants’ age and socioeconomic situation, as well as the intervention type. Type of methodology will not be included in the search terms in order not to lose studies in which terms related to RCTs were not used in articles titles or as keywords. The first search will be conducted in the Web of Science core collection and will subsequently be piloted in the other databases, adapting the search depending on the databases’ possibilities and limitations. Title and abstract will be selected where feasible. A final search will be piloted prior to the systematic review publication, ensuring an update of studies that should be included in the review. The search will not be restricted by language limits. The reference lists of relevant systematic reviews will be screened for potential articles to include.

### Data collection and analysis

#### Study selection

The studies will be gathered using the Mendeley platform and later will be organized in an excel file to proceed with the elimination of duplicates and selection of suitable studies. After eliminating duplicate entries, two reviewers will conduct the title and abstract screening, deciding independently if each study meets the inclusion criteria. Each study will be classified as included or excluded. At this point, researchers will not exclude studies if some key indicators are not specified in the abstract (e.g., RCT methodology, socioeconomic indicator). References to relevant studies in languages other than English or Spanish will be listed as “pending” in the review in order to include potentially relevant studies that the review team is not able to translate. In cases where the classification decision is not immediately obvious, the study will be classified as included. Next, both reviewers will independently assess each selected study’s full text in order to evaluate the relevance to the systematic review. In this step, exclusion reasons will be compiled. Finally, review authors will compare the classifications, and a third reviewer will participate in the process in case of disagreement. The level of agreement between researches will be calculated using the Kappa index [56].

#### Data extraction and management

Data extraction will be done independently by two reviewers. As per the classification phase, a third reviewer will intervene if consensus cannot be achieved. In cases where there is missing data which is required to classify the study, the original authors will be contacted in order to obtain the information necessary to complete the classification. In case a relevant study is not found by this via, hand-searching will be conducted.

The following qualitative data should be collected: author, year of publication, country, age/sex, race/ethnicity, sample (control/intervention group), SES indicator, cognitive domain, intervention tool/programme, intervention period, timing (frequency-duration per session), session format (individual/group), intervention conditions, intervention providers, co-interventions, outcome (assessment tool), and follow-up measures. Data will be summarised in a table as well as in the results section. Due to the variety of outcomes expected and the likely heterogeneity among samples and population traits, review authors will carry out a synthesis of the data as is explained in the “Synthesis of results” section.

### Assessment of risk of bias

A bias assessment will be done by two reviewers with disagreements resolved by a third reviewer. In order to assess the internal validity of the selected studies, reviewers will use the Revised Cochrane risk-of-bias tool for RCTs (RoB 2) [53, 57]. This tool is useful to identify the likely bias that may specifically be affecting the RCTs results due to the use of different sources. The five domains that are revised via this tool are bias (1) arising from the randomization process, (2) due to deviations from intended interventions, (3) due to missing outcome data, (4) in measurement of the outcome, and (5) in selection of the reported result.

The previously mentioned tool includes different statements in each domain. Each reviewer will judge each article as “low risk of bias”, “some concerns”, or “high risk of bias”. The overall risk level of the article will be a composite of the responses in each individual domain. If each single component of each domain is considered as having “low risk of bias”, then the domain will be considered “low risk of bias”. In order to classify those cases in which “low risk of bias” and “some concerns” exist throughout domains, the final judgement for the article will be “some concerns”.

Moreover, to rate the quality of scientific evidence in the systematic reviews the Grading of Recommendations Assessment, Development and Evaluation (GRADE) system will be used. The GRADE system helps to determine if the estimated effect size is reliable or not as defined according to four categories: high, moderate, low, and very low [58].

### Synthesis of results

The level of heterogeneity that exists among the selected studies (e.g., because of the different cognitive domains targeted for intervention, range of years used as inclusion criteria, outcome measures, instruments) will determine if a meta-analysis can be feasibly conducted. In the case where the data are amenable to a meta-analysis being used, data will be ordered in an excel document and the Comprehensive Meta-Analysis software package (CMA) will be used to transform data into a suitable format. Effect sizes will be calculated using standardized mean differences (SMD) and 95% confident intervals (CIs). The SMDs will be interpreted as per Cohen’s guidelines [59], with values of 0.2, 0.5, and 0.8 being considered as small, medium, and large effect sizes, respectively. Since it is expected that samples will be vary in number and characteristics, a random effects model will be used in order to control the heterogeneity. To assess heterogeneity, reviewers will use forest plots, *p* values, and *I*^2^ index values with 95% CIs. *I*^2^ index values for heterogeneity expressed in percentages will be interpreted as: minor (0–40%), moderate (30–60%), substantial (50–90%), and considerable (75–100%) [60]. Where possible, a sub-group analysis will be conducted in order to explore the source of heterogeneity across the included studies.

In case the data are not suitable to allow a meta-analysis to be conducted, a synthesis of the data will be carried out by following the Synthesis Without Meta-analysis (SWiM) in systematic reviews guidelines [61]. The SWiM approach is recommended in situations where the heterogeneity of the data makes a meta-analysis difficult to implement; for example, due to the diversity of the studies in terms of, for example, measures used to estimate the effect size, intervention types, outcomes, etc.

Considering the aim of the systematic review, the intervention articles will be grouped in terms of the cognitive domain involved (e.g., studies that are focused on language development, executive functions, memory). In order to offer a description of the standardized metrics used in the studies, the different methods used by each study’s authors will be analysed, and the studies will be subsequently grouped by these factors (e.g., mean differences, *p* values). The selection of the method will be justified.

The synthesis of results will include studies which report any degree of risk of bias, since the main objective of the systematic review will be to offer a comprehensive analysis of what has been done in the cognitive intervention field. This limitation will be highlighted in the limitations section of the systematic review. Limitations of the synthesis of results will be provided, reporting how the selected groupings formed in interpreting the data can influence in the response to the systematic review question. Furthermore, the method to examine heterogeneity (e.g., harvest plots, median effect sizes) will be selected, if relevant, based on the characteristics of the studies included.

## Discussion

Worldwide, approximately 650 million children live in poverty or low-SES conditions. Low SES has been shown to negatively influence processes related to physical, emotional, and cognitive development [8, 9]. Thus, interventions designed to improve cognitive development in children living in low-SES conditions would potentially greatly benefit these children [62]. Nevertheless, to the best of our knowledge, no systematic review of such studies has been previously undertaken. Consequently, we set out to design a protocol for performing such a review.

The protocol described here contributes to the field considering that it is: (1) including literature regardless of year of publication, (2) assessing risk of bias of the studies through a standardized tool, (3) taking into account a wide age range in the populations who participated in interventions, (4) including interventions in any type of cognitive domain, which will give to the reader a comprehensive understanding of the field, and (5) only including RCT studies, which are the gold standard to test intervention efficacy. Furthermore, any systematic review based on this protocol would offer a comprehensive approach for a population that could benefit from an intervention undertaken by experts and agents involved in childhood developmental issues.

Among other strengths, the systematic review will offer results obtained through a methodical search in diverse databases. Moreover, review authors will hand-check the relevant literature contained in the references and other sources of interest.

Nevertheless, one limitation of the study is that the inclusion criteria are quite wide (e.g., age: children from 0 to 17; interventions undertaken in any cognitive domain). Thus, the selected studies will probably be heterogeneous, and consequently, it may be difficult to assimilate the data in order to undertake a quantitative analysis. Due to this possibility, the objective of the protocol will be to offer an al alternative approach which does not require the quantitative comparison of studies. Another limitation of this review could be the exclusion of studies using other kind of methodologies (that is, non RCTs designs), as other designs could be also be suitable for assessing the outcomes of interest. Although the quasi-experimental methodology is widely used in this field of study, it will be excluded by this protocol as we are interested in including the most robust, reliable studies in the review. Accordingly, the systematic review may be missing effective cognitive interventions for children from low-SES settings. Nevertheless, the inclusion of other kinds of methodologies could be considered as an extension to the proposed protocol to fully understand what has been effectively implemented so far.

The systematic review will be useful as it will offer a framework based on evidence, to organisations, institutions, and experts who want to implement or promote interventions aimed at enhancing cognitive domains in children and adolescents who live in disadvantaged contexts. Consequently, this study could inform interventions and policy settings, such as those that need to achieve the Sustained Developmental Goals (SDGs) about children education included in the 2030 Agenda. The fact that the systematic review will be composed only by RCTs will support the conclusions based on effectiveness criteria, capital aspect to policy decision making. This review will also provide researchers with a summary of what has proven to be effective depending on various intervention factors and sample features, offering future lines of research in the field where debate is still open.

## Supplementary Information

**Additional file 1: PRISMA-P checklist**. PRISMA-P checklist for systematic review protocol.

**Additional file 2.** Search strategy. Initial search strategy used in Web of Science Core collection.

## Data Availability

Not applicable.

## Abbreviations

ADHD: Attention deficit hyperactivity disorder
RCT: Randomized controlled trials
SES: Socioeconomic Status
SDGs: Sustainable Developmental Goals
UN: United Nations
UNICEF: United Nations Children’s Fund
WoS: Web of Science
ERIC: Education Resources Information Center
GRADE: Grading of Recommendations Assessment, Development and Evaluation
