# Supplementary material for: Cognitive interventions in children and adolescents from low socioeconomic status backgrounds: a systematic review protocol of randomized controlled trials
Source: Syst Rev. 2021 Jun 25;10:187. doi: 10.1186/s13643-021-01738-x (PMC8235624; doi:10.1186/s13643-021-01738-x)
Supplement: Supplementary file 2 — Additional file 2. Search strategy. Initial search strategy used in Web of Science Core collection. [file 13643_2021_1738_MOESM2_ESM.docx]

Cognitive interventions in children and adolescents from low socioeconomic status backgrounds: A systematic review protocol of randomized controlled trials

**Additional file 2**

**Search strategy:**

Search applied to Web of Science (WoS) Core collection:

**To identify population (Title/abstract):**

1. child*

2. adolescen*

3. teen*

4.youth*

5. “school student*”

6. infant*

7.“pre-school student”

8. kindergarten

9. baby

10. toddler*

11. 1 OR 2 OR 3 OR 4 OR 5 OR 6 OR 7 OR 8 OR 9 OR 10

**To identify socioeconomic status of the population (Title/Abstract):**

12. income*

13. socioeconom*

14. econom*

15. exclusion

16. poverty

17. 12 OR 13 OR 14 OR 15 OR 16

**To identify different types of cognitive interventions (Title/Abstract):**

18. (cogniti* NEAR/3 stimulat*)

19. (cogniti* NEAR/3 training)

20. (cogniti* NEAR/3 intervent*)

21. (cogniti* NEAR/3 enhanc*)

22. 18 OR 19 OR 20 OR 21

23. 11 AND 17 AND 22
